# Testing hypotheses for maternal effects in *Daphnia magna*

**DOI:** 10.1111/jeb.13206

**Published:** 2017-11-22

**Authors:** C. M. Coakley, E. Nestoros, T. J. Little

**Affiliations:** ^1^ Institute of Global Change School of GeoSciences University of Edinburgh Edinburgh UK; ^2^ Institute of Evolutionary Biology School of Biological Sciences University of Edinburgh Edinburgh UK

**Keywords:** fecundity, matching environments, maternal age, maternal effects, transgenerational

## Abstract

Maternal effects are widely observed, but their adaptive nature remains difficult to describe and interpret. We investigated adaptive maternal effects in a clone of the crustacean *Daphnia magna,* experimentally varying both maternal age and maternal food and subsequently varying food available to offspring. We had two main predictions: that offspring in a food environment matched to their mothers should fare better than offspring in unmatched environments, and that offspring of older mothers would fare better in low food environments. We detected numerous maternal effects, for example offspring of poorly fed mothers were large, whereas offspring of older mothers were both large and showed an earlier age at first reproduction. However, these maternal effects did not clearly translate into the predicted differences in reproduction. Thus, our predictions about adaptive maternal effects in response to food variation were not met in this genotype of *Daphnia magna*.

## Introduction

The environment, condition or phenotype of a mother can account for a significant amount of variation in the traits of her offspring (Wilson *et al*., 2005). Such maternal effects are known for immunity (Coakley *et al*., 2014), variation in feeding rate (Garbutt & Little, 2014), anti‐predator behaviour (Agrawal *et al*., 1999) and dispersal traits (Dingle, 2014), among others. Maternal effects appear to be important across a wide range of organisms including mammals (Glezen, 2003), invertebrates (Stjernman & Little, 2011), fish (McGhee *et al*., 2012), birds (Boulinier & Staszewski, 2008) and plants (Vivas *et al*., 2015). Theoretical studies have shown the strong potential of maternal effects to alter population dynamics (Ginzburgh, 1998) and population genetic structure (Wade, 1998; Wolf *et al*., 1998), and so ultimately the evolutionary potential of a population (Kuijper & Hoyle, 2015). Yet, in most cases, it is unclear how, or even if, maternal effects are adaptive (Mousseau & Fox, 1998; Marshall & Uller, 2007).

It is hypothesized that maternal effects are a successful adaptive strategy in variable, but predictable environments. In such cases, mothers can integrate information about the environment, or their condition, to produce offspring with traits that confer high fitness in the expected conditions. This predictability, that is for anticipatory maternal effects (Marshall & Uller, 2007), can take two forms: when there is a positive environmental correlation across a generation, mothers prepare their offspring for an environment similar to their own, while under negative correlations across a generation, mothers prepare their offspring for the opposite environment (Kuijper & Hoyle, 2015). Light sensitivity in plants provides an example of the former: *Campanulastrum americanum* from mothers of either light gap or understory environments do better in that matched environment (Galloway & Etterson, 2007). Growth rate of *Caenorhabditis elegans* under normoxic and anoxic environments is an example of a negative correlation (Dey *et al*., 2016). It is often difficult to determine the adaptive nature of transgenerational plasticity in matched or unmatched environments due to the presences of ‘silver spoon’ or carry‐over effects (Engqvist & Reinhold, 2016), which may mask truly adaptive benefits to offspring of being in a matched environment. Indeed, outside of a handful of well‐known examples (Galloway & Etterson, 2007; Merrill & Grindstaff, 2015), adaptive maternal effects have proven difficult to demonstrate (Uller *et al*., 2013).

It is well established that older mothers produce offspring of different quality to younger mothers (Moorad & Nussey, 2016). For example, in some species, offspring of older mothers are larger at birth, mature to a greater size and show greater early‐life reproduction which might trade‐off with longevity and lifetime reproductive success (Metcalfe & Monaghan, 2001; Priest *et al*., 2002; Benton *et al*., 2008; Plaistow *et al*., 2015). Similar observations have been made in our study species, the crustacean *Daphnia magna,* where we have observed that increasing maternal age is linked to increasing size at birth, enhanced parasite resistance and changes in reproduction (Clark *et al*., 2017). Variation in maternal nutrition in *D. magna* appears to produce similar phenotypes, as the offspring of dietary restricted mothers produce relatively large, parasite resistant offspring (Garbutt & Little, 2017). These increases in body size in offspring from dietary restricted mothers may be adaptive if these mothers can expect their offspring to be born into a low food/high competition environment, and assuming that large offspring have an advantage in this circumstance. Older mothers tend to live in more competitive environments, as might be expected further into the growing season of seasonal organisms, and here again larger offspring could be advantageous.

This study explores adaptive maternal‐effect hypotheses; specifically, we aimed to determine how both maternal food and maternal age impact offspring performance in a clone of *D. magna*. We subjected mothers to plentiful food or to dietary restriction and took offspring from clutch one, two or five (to create different age classes). These offspring were placed under plentiful food or dietary restriction, and their reproductive performance measured. Our predictions are as follows: in a food environment matched to their mothers, offspring will perform better in their reproduction compared to those in unmatched environments (in line with anticipatory maternal effects theory). Our second prediction is that offspring of older mothers (e.g. individuals from clutch five) will reproductively out‐perform the offspring of younger mothers in food‐restricted environments. As we studied both maternal age and dietary restriction simultaneously, we also explore the interaction effects of these factors.

## Materials and methods

This study used a single clone of *D. magna* collected from the Kaimes population in the borders of Scotland that has been the subject of numerous maternal effects investigations (see (Mitchell & Read, 2005; Stjernman & Little, 2011; Garbutt & Little, 2014; Clark *et al*., 2017). The particular clone chosen displays the typical response of this population to environmental stresses. The use of a single clone enhances our power to disentangle the studied effects, as this minimizes variation arising from genetic difference (Little & Colegrave, 2016).

**Figure 1:**
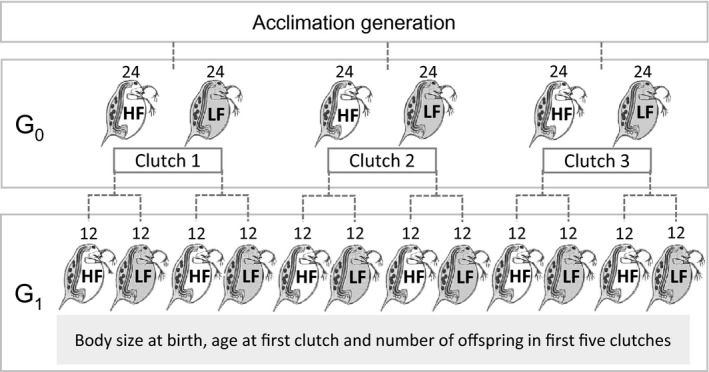
G_0_ represents the maternal generation for the main analysis. Maternal age is the G_0_ clutch that G_1_ was born from (either early – clutch 1, mid – clutch 2 or later life – clutch 3). G_0_ individuals were given either ad libitum (HF) or restricted low (LF) food; therefore, the G_0_ generation has two treatment types: food and age. The offspring generation (G_1_) was given HF or LF; measurements were recorded regarding their body size at birth and reproductive performance (age at first clutch and number of offspring produced). Numbers above *Daphnia* indicate sample size at each stage.

To control the effect of any pre‐existing transgenerational effects, 48 replicates, each an individual *Daphnia* in a 60‐ml jar, were maintained under *ad libitum* food conditions (8.75 × 10^6^ *Chlorella* algae per day) and standardized to at least three generations (acclimation generations). Two individuals from the third brood of each clonal lineage were chosen at random and subjected to either *ad libitum* food (8.75 × 10^6^ *Chlorella* algae per day) or dietary restricted (1.75 × 10^6^ *Chlorella* algae per day) environments within 18 hr after birth (this is the G_0_ generation), to give a total of 96 individuals. This number of individuals is higher than needed as deaths were anticipated. Taking 96 individuals ensured at least 72 lines, which was required for our study. Again, and throughout, individuals were housed singly in 60‐mL jars, which were stored in climate chambers at 20°C with 16 h of light and 8 h of dark per day. Two offspring were taken from the first clutch of 24 of 72 G_0_ mothers, and each of these G_1_ offspring was exposed to one of the two dietary treatments. Two offspring were also taken from the second clutch of a further 24 (i.e. not the same 24 mothers that contributed first clutch offspring) G_0_ mothers, and each of these G_1_ offspring was again exposed to one of the two dietary treatments. Two final offspring were taken from the fifth clutch of a further 24 (not the same mothers that contributed first or second clutch offspring) G_0_ mothers, and each of these G_1_ offspring was again exposed to one of the two dietary treatments. In total then, there were 144 G_1_ offspring (See Figure 1).

The clutch that an individual came from (first, second or fifth) was used as a proxy for maternal age and was considered as an explanatory variable (Figure 1). The use of clutch as a proxy for age allowed us to compare a treatment group's biological age rather than chronological age.

The body size at birth of every G_1_ individual was measured, using a camera and imagej software within 18 h from birth. The later reproductive performance of these (G_1_) individuals was measured as age at first reproduction and number of offspring in the first five clutches.

### Statistical analysis

Age at first reproduction is a ‘time to event’ variable and was thus subject to a Cox proportional hazards analysis. We provide risk ratios and their confidence limits for the age at first reproduction analysis. The other response variables, number of offspring born in the first five clutches and body size at birth, were analysed with anova, which included all possible interactions between our explanatory variables. A breakdown of all the models explored can be found in the Appendix S1. The number of offspring born in the first five clutches was square‐root‐transformed to meet the assumptions of normality. The explanatory variables were maternal food, maternal age and offspring food (although not for G_1_ size at birth, as this would not be relevant). For all anova, we provide effect sizes (*η* ^2^) in addition to test statistics and *P*‐values. All analyses were performed using jmp software (Version 12.1.0) with the default (for anova) implementation of type III sum of squares. We used a backward elimination process for all analysis and excluded interactions terms with *P *> 0.05.

## Results

### Maternal effects on offspring body size

**Figure 2:**
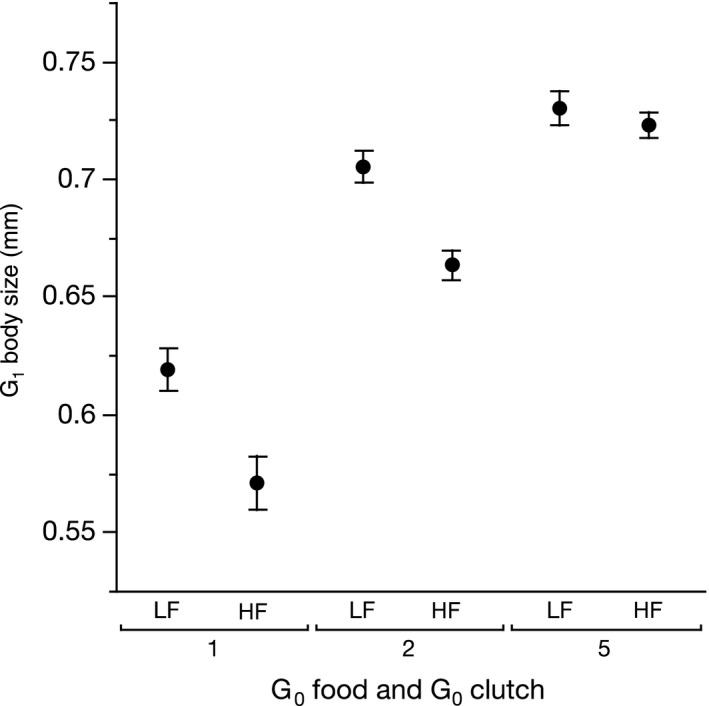
(A) The effect of mothers (G_0_) food and age (defined by clutch) on offspring body size (G_1_ generation). Error bars represent one standard error around the mean. LF indicates low maternal food, and HF indicates high maternal food.

G_1_ body size at birth was influenced by maternal (G_0_) food (F_1,118_ = 27.3, *P* < 0.0001, *η* ^2^ = 0.064), maternal age (F_2,118_ = 142.7, *P* < 0.0001, *η* ^2^ = 0.63) and their interaction (F_2,118_ = 4.6, *P* = 0.012, *η* ^2^ = 0.016). Offspring body size increased with maternal age, and offspring of low food mothers were larger in the first two clutches, but a maternal food effect was not evident in the oldest mothers (Figure 2).

### Fecundity

**Figure 3:**
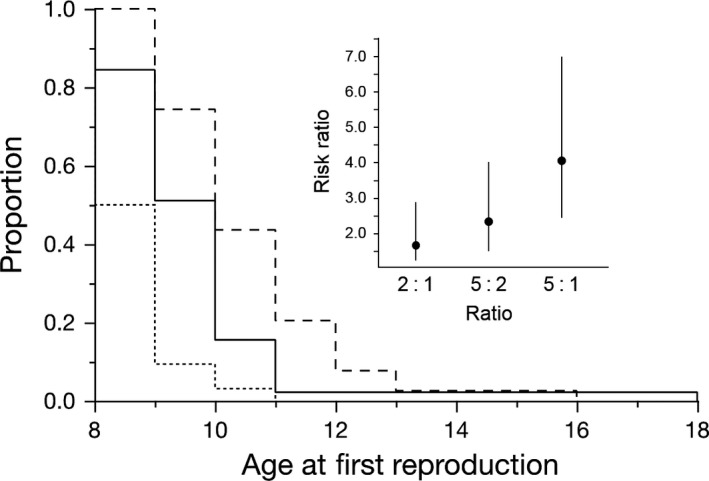
Step series graph of the effects of age on time to age at first reproduction (G_1_ generation). Dotted line represents the oldest age group (clutch 5), solid line represents the middle age group (clutch 2), and the dashed line represents the youngest group (from clutch 1).

There was no significant interaction between G_0_ food and G_1_ food on age at first reproduction, nor was there a main effect of G_0_ food. G_1_ age at first reproduction depended on the food they were given, that is G_1_ food (*X* ^2^ = 17.7, *P* = < 0.0001), with well‐fed *Daphnia* reproducing earlier. Age at first reproduction also showed a significant relationship with maternal age (*X* ^2^ = 21.3, *P* < 0.0001; Figure 3), where individuals from older mothers started reproduction early. Hazard ratios and their confidence limits for this proportional hazards analysis are shown in the Figure 3 inset. No significant effect of a maternal age was noted for the timing of later clutches. Maternal age did not interact with other factors.

**Figure 4:**
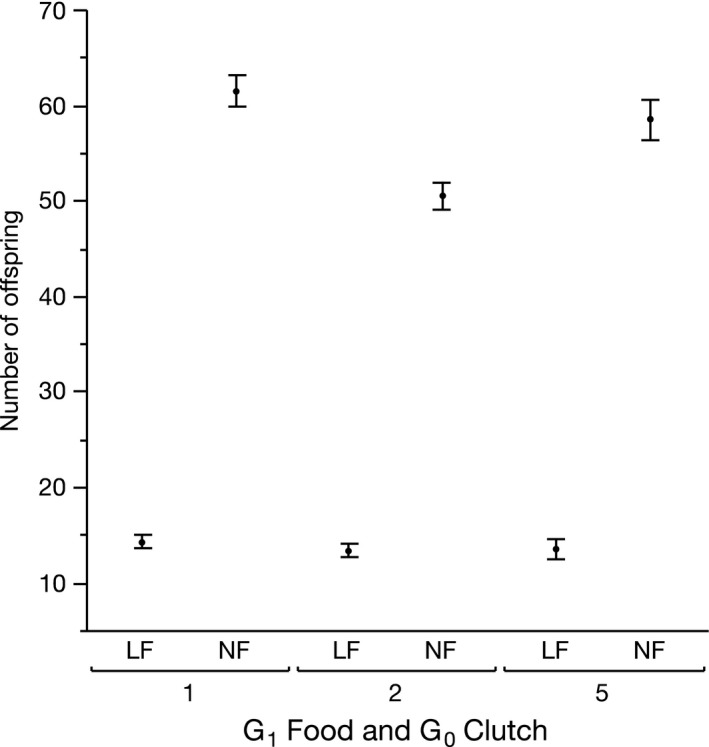
Total number of offspring produced by (G1) *Daphnia* depending on the food they receive and the age of their mother (defined by G_0_ clutch). Error bars represent one standard error around the mean. LF indicates low food of G_1_, and HF indicates high food of G_1_.

The number of offspring in the first five clutches (Figure 4) was largely explained by G_1_ food (individuals under low food produced significantly fewer offspring: F_1,109_ = 1740, *P* < 0.0001, *η* ^2^ = 0.91), but also maternal age (F_2,109_ = 8.67, *P *= 0.000, *η* ^2^ = 0.009) and an interaction between G_1_ food and maternal age (F_2,109_ = 4.6, *P* = 0.012, *η* ^2^ = 0.005; Figure 4). No significant interaction between G_0_ and G_1_ food was detected. We also performed a limited analysis of grandmaternal effects and present this as Appendix S1.

## Discussion

In this study, we investigated two maternal effects (maternal age and food) on measures of offspring performance in a clone of the fresh water crustacean (*Daphnia magna*). Our first prediction was that offspring in a food environment matched to their mothers should show greater reproductive performance. However, we found no benefit to being in a food environment matched to your mother. Our second prediction was that offspring of older mothers would fare better in low food environments. We found evidence for maternal age effects on age at first reproduction, which may be adaptive. However, this potential adaptive maternal effect was not specifically in line with our prediction, which required a significant maternal age by offspring food interaction to be met. For the other measure of reproductive performance, the number of offspring produced in five clutches, we found very weak effect sizes, with the direction of effects being counter to predictions. Thus, we conclude that there is only weak evidence for adaptive maternal effects in this study. We also conclude that size at birth, whether determined by maternal food or maternal age, does not have straightforward effects on subsequent reproductive success.

## Matched and unmatched food environments

We did not observe maternal food by offspring food interactions for any traits, and thus, the basic prediction of adaptive maternal effects theory was not met. Moreover, a large body size as a consequence of low maternal food had no downstream performance advantages in the *Daphnia* clone we studied. Food availability fluctuates in the *Daphnia* environment (Murdoch *et al*., 1998; McCauley *et al*., 1999), and thus, maternal effect driven by maternal food is a realistic prediction. However, offspring food clearly, and unsurprisingly, played the most important role in all traits. This is similar to the findings of a meta‐analysis (exploring both plants and animals), which revealed subtle effects of matching environments compared to the direct effects of the focal environment (Uller *et al*., 2013). It is possible that low maternal food is a not a cue for future maternal food, but is instead a cue for other threats, such as the increased infection risk associated with crowding (Clark *et al*., 2017); see also (LaMontagne & McCauley, 2001). As seen in another study (Beyer & Hambright, 2017) when making predictions about the adaptive significance of maternal effects, it will, in many cases, be difficult to know exactly what mothers are preparing their offspring for, and that the basic idea of matching environments will often be too simplistic.

## Maternal age effects

In food‐restricted environments, we predicted that the large offspring of older mothers would show better reproductive performance compared to offspring of younger mothers. This prediction was not wholly met: significant maternal age effects on offspring age at first reproduction were detected, but these were observed in both offspring food environments. Nonetheless, the effect of maternal age on offspring age at first reproduction was substantial (Figure 3). Age at first reproduction is an important component of reproductive performance (Forslund & Pärt, 1995; Krüger, 2005) and should be particularly important for *D. magna,* where populations can increase dramatically over a season, and early reproduction secures resources for offspring over competitors. However, this timing of reproduction did not seem to lead to differences in the total number of offspring produced (Figure 4). Indeed, effect sizes for the influence of maternal age on number of offspring were notably small (the significant maternal age by offspring food interaction explained less than 1% of variance). Whereas our study found limited effects of these traits for a single mother's reproductive success, there could be a significant impact on subsequent population dynamics. Differences in age at first reproduction or offspring size, particularly for a short‐lived species such as *D. magna,* could result in differences in competitive environments of the next generation. This in turn could benefit some individuals more than others (via maternal effects and environmental conditions) for that generation, as seen in a study exploring maternal effects and population dynamics in *Sancassania berlesei* (Benton *et al*., 2005).

Within the *Daphnia* system, as with low food, older mothers are established to produce offspring that are less susceptible to infection (Clark *et al*., 2017) and thus aged mothers may be preparing their offspring for a harsh environment. Presently, this appears to be specific to the threat of parasitism rather than food stress. Although there was substantial genetic variation for this maternal effect in *Daphnia*, the average effect was for high resistance in offspring from poorly fed mothers (Stjernman & Little, 2011). Elsewhere, maternal age effects have been found to impact offspring size in *Lemna minor* (Barks & Laird, 2016), offspring development and maturation size of *S. berlesei* (Benton *et al*., 2008) and early‐life reproduction of *Daphnia* (Plaistow *et al*., 2015). It is thus important to note that there is the potential for maternal age to be adaptive for traits or environments that we did not explore. For example, considering competitive ability, as opposed to the performance proxies we used, might paint a different picture, as seen in a study using *S. berlesei* (Benton *et al*., 2005). In addition, different populations will face different environmental pressures, and the occurrence of adaptive maternal effects could well differ between populations (Vijendravarma & Kawecki, 2015; Walsh *et al*., 2016).

Only a handful of studies have explored multiple maternal effects, as we did. Maternal age and food effects were explored in seed beetles (*Callosobruchus maculatus*) (Fox & Dingle, 1994), but these beetles show different patterns from those observed presently. For example, older adult beetles produced small offspring that developed slowly, the opposite of what we observed in *D. magna*. Older or poorly fed yellow dung flies (*Scathophaga stercoraria*) also produce smaller eggs that then perform poorly (Jann & Ward, 1999). In our experiment, the age of *Daphnia* mothers interacted with maternal food, specifically, maternal food effects appeared dampened in older mothers (Figure 2).

## Conclusions

Although we detected numerous maternal effects, most notably the large size of offspring from poorly fed or older mothers and the early age at first reproduction of offspring born to older mothers, the adaptive nature of these effects were not clear. Other genotypes, or other traits, might respond differently to our treatments. At the same time, the production of larger offspring with different reproductive features would itself alter the competitive environment (Beckerman *et al*., 2006; Kindsvater *et al*., 2011; Prior *et al*., 2011), a scenario that could more fully reveal the consequences of maternal effects.

## Supporting information


**Appendix S1** Maximal models and additional analysis.
