# Supplementary material for: Testing hypotheses for maternal effects in Daphnia magna
Source: J Evol Biol. 2017 Nov 22;31(2):211–6. doi: 10.1111/jeb.13206 (PMC6849578; doi:10.1111/jeb.13206)
Supplement: Supplementary file 1 — Appendix S1 Maximal models and additional analysis. [file JEB-31-211-s001.docx]

**Supplementary Material**

Maximal Models from manuscript

**Maternal effects on body size**

**Maternal age and food effect on offspring body size**

G1 body size ~ G0 age * G0 food

**Fecundity measures**

**Maternal treatment effects and current food environment on age at first reproduction**

G1 age at first reproduction ~ G0 age * G0 food * G1 food

**Maternal treatment effects and current food environment on total reproduction**

G1 total reproduction ~ G0 age * G0 food * G1 food

Additional Analysis

**Grand maternal effects on body size**

**Grand maternal age effect and maternal age and food effect on offspring body size**

G2 body size ~ G0 age * G1 age * G1 food

**Results (Grand maternal effects on body size)**

We also studied if size at birth was determined by grandmaternal age effects, by exploring the body size at birth of the offspring of G1 individuals (i.e. G_2_ size at birth). G_2_ body size at birth was influenced by maternal (G_1_) food (F_1,103_ = 11.86, p = 0.0008, χ^2^ = 0.087), and an interaction between G_1_ food and grandmaternal age (F_2,103_ = 5.45, p = 0.006, χ^2^ = 0.080). G_2_ offspring body size was significantly larger in the first two grandmaternal age classes and when mothers were under low food, however this effect of food disappears in the oldest age class (Figure A1), as seen with the previous analysis (Figure 2). There was no significant interaction between G_1_ food and G_0_ food on G_2_ body size.

**
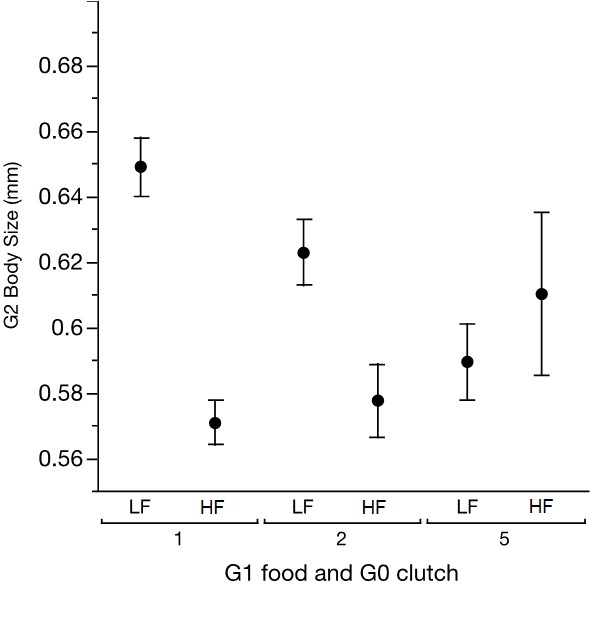
**

**Figure A1** The effect of maternal food (G_1_) and grandmaternal age (G_0_) on offspring body size (G_2_). Error bars represent one standard error around the mean. LF indicates low maternal food, HF high maternal food.
